# The synergistic impact of "SO-TO-DO" and one-minute preceptor models on skill and knowledge development

**DOI:** 10.6026/973206300210753

**Published:** 2025-04-30

**Authors:** Charushila Rukadikar, Sangeeta Gupta, Arani Das, Anil Gangwar, Avinash Singh, Shaista Saiyad, Neeraj Mahajan, Atul Rukadikar

**Affiliations:** 1 Department of Physiology, AIIMS Gorakhpur, Uttar Pradesh, India; 2 Department of Physiology, KGMU Lucknow, Uttar Pradesh, India; 3 Department of Orthopaedic, SGRRIM & HS, Patel Nagar, Dehradun, Uttarakhand, India; 4 Department of Physiology, Smt. NHL Municipal Medical College Ahmedabad, Gujarat, India; 5 Department of Microbiology, AIIMS Gorakhpur, Uttar Pradesh, India

**Keywords:** Competency-based medical education (CBME), hybrid learning model, one-minute preceptor (OMP), skill acquisition, medical training, student perception

## Abstract

Medical education transforms to competency-based systems which require knowledge with practice. Hence, integrating "So-to-Do" with Modified One-Minute Preceptor (OMP) model is critical. First-year medical students showed better retention of learning material and better accuracy in device operation by using this combined method for sphygmomanometer and audiometer and ECG. The new assessment pattern involving MCQs and OSPE delivered better results than conventional instructional methods. Evaluation from students along with faculty demonstrated higher levels of participation while showing better understanding among participants alongside enhanced evaluative abilities. Students learned their skills better because of structured feedback given in a stepwise manner. Proof from the hybrid teaching approach indicates its ability to deliver efficient modern medical education systems.

## Background:

Medical education has changed dramatically over the past century. What began as an apprenticeship-based model, where students learned by observing experienced physicians, has evolved into a structured, competency-driven approach that emphasizes not just knowledge, but real-world application [1]. Competency-Based Medical Education [CBME] is now at the forefront of this evolution, designed to ensure that medical graduates possess not only theoretical knowledge but also the practical skills and professional behaviours required for patient care [2, 3]. Unlike traditional methods that rely on classroom learning and exams to assess understanding, CBME prioritizes skill-based, hands-on learning, where student's progress based on demonstrated competence rather than time spent in training [4]. A major advantage of CBME is its emphasis on direct observation and feedback, allowing educators to identify gaps early and provide targeted support to learners [5, 6]. However, while CBME has improved medical training in many ways, its success relies heavily on effective teaching strategies that integrate both knowledge acquisition and skill development in a seamless and engaging manner [7, 8]. Despite this shift, traditional teaching methods still struggle to bridge the gap between theoretical learning and clinical application. In many medical schools, knowledge and skills are taught separately, with the first few years focused on lectures and exams, while hands-on experience comes much later [9, 10]. As a result, students may excel in theory but feel unprepared when faced with real patients. One long-standing teaching philosophy, often summed up as "see one, teach one, do one," assumes that students can perform a procedure after watching it just once [11, 12]. While this approach has been a rite of passage for generations of doctors, it lacks structured guidance and feedback, often leading to inconsistent learning experiences and in some cases, compromising patient safety [13, 14]. Educators have recognized that more structured, feedback-rich approaches are needed to ensure that students not only memorize procedures but also develop the confidence and competence to perform them independently [15, 16].

The "So-To-Do" model is a structured approach to skill acquisition that provides a step-by-step learning progression, particularly effective for mastering complex or multi-step clinical procedures [17]. It consists of four key phases: See, where students build a theoretical foundation through demonstrations, lectures, or videos; Observe, where they watch experts perform the skill in real or simulated clinical settings to bridge theory and practice [18]; Train, where they practice under supervision, receiving real-time feedback to refine their technique [19, 20] and Do, where they independently perform the skill, applying their knowledge and training in a clinical setting [21, 22]. By progressing through these phases, students build confidence gradually, ensuring they are not just imitating actions but truly understanding each step. Studies show that stepwise models like Peyton's Four-Step Approach, which follows a similar structured progression, lead to higher skill retention and greater procedural accuracy compared to traditional "watch and do" methods [23, 24]. While stepwise learning is valuable, it alone is not sufficient, as students may struggle with decision-making and clinical reasoning in real-world situations without proper feedback and critical thinking development [25, 26]. This is where the One-Minute Preceptor (OMP) model complements the So-to-Do approach. The OMP method is a highly effective micro-teaching strategy that allows instructors to provide structured feedback and reasoning-based learning within a short time [27, 28]. The model consists of five micro skills: Get a commitment, where students state their thoughts on a case or procedure; Probe for supporting evidence, encouraging them to explain their reasoning; Teach a general principle, providing relevant rules or guidelines to reinforce learning; Reinforce what was done well, highlighting positive aspects of their performance; and Correct mistakes, offering constructive feedback to address errors and enhance understanding [29, 30].

Appropriately delivered feedback in medical education aligns with best practices and is valued by learners [31]. Research has shown that OMP-based feedback improves clinical reasoning skills, confidence and learner engagement, making it a valuable addition to skill-based teaching [32, 33]. Given the strengths of both models, integrating the So-to-Do and OMP techniques into a single hybrid teaching strategy offers a more holistic and effective approach to medical training. The So-to-Do method ensures procedural competence, allowing students to practice skills step by step with structured supervision. Meanwhile, the OMP method enhances clinical reasoning and feedback, ensuring that students not only know how to perform a procedure but also understand when and why to use it [34, 35]. Therefore, it is of interest to combine these methods, so as to create a learning experience that is engaging, interactive and evidence-based, ultimately producing more competent and confident healthcare professionals.

## Methodology:

This cross-sectional study was conducted among first-year MBBS students at the All India Institute of Medical Sciences (AIIMS), Gorakhpur, Uttar Pradesh to evaluate the effectiveness of a hybrid teaching model in improving theoretical knowledge and practical skills. Ethical approval for the study was obtained from the Institutional Human Ethics Committee (IHEC), AIIMS Gorakhpur (Approval No: IHEC/AIIMS GKP/BMR/83/2022) and all procedures followed the ethical guidelines outlined by the institution. Before participation, students were individually counselled regarding the study's objectives and methodology. Written informed consent was obtained, ensuring voluntary participation. Confidentiality was maintained throughout, with participant data anonymized and used solely for research purposes. The study enrolled a total of 126 first-year MBBS students, selected based on specific inclusion and exclusion criteria. Only those currently enrolled in their first year were considered eligible. Students who had prior formal training in using an ECG device, audiometer, or sphygmomanometer or were unwilling to participate were excluded. The selection process ensured that all participants had a similar baseline level of competency, enabling a fair and standardized evaluation of the hybrid learning model as mentioned in Table 1.

## Results:

To enhance skill acquisition, students were trained in the use of a sphygmomanometer, audiometer and ECG device through a structured hybrid teaching approach that integrated two well-established methodologies: the "See One, Teach One, Do One" (SO+TO+DO) method and the one-minute preceptor (OMP) model. The hybrid model aimed to create a progressive learning experience, combining hands-on practice with structured feedback. The methodology was validated by medical education experts, ensuring its effectiveness in competency-based medical training. Students were guided through a stepwise learning process that incorporated multiple stages to reinforce both theoretical understanding and skill proficiency. The learning began with the observation phase, where students watched an expert demonstration of each procedure. This phase allowed them to familiarize themselves with the workflow, instrument handling and key procedural steps. Following this, the explanation phase provided an opportunity for faculty members to break down the procedure in detail, covering scientific principles, clinical relevance, potential errors and best practices. After the theoretical groundwork was established, students moved on to the performance phase, where they performed the procedure under direct faculty supervision. This hands-on experience was crucial in helping students transition from passive observers to active practitioners, reinforcing procedural accuracy. During the critical reasoning and information retrieval phase, faculty engaged students in real-time questioning, encouraging them to justify their procedural choices and apply evidence-based reasoning. This phase was instrumental in developing clinical decision-making skills alongside procedural proficiency. The feedback phase ensured that students received immediate, structured guidance on their performance, allowing for the correction of errors in real time and reinforcing correct procedural techniques. Lastly, in the peer teaching phase, students who had successfully learned the skill were encouraged to teach their peers. This approach not only strengthened their-own understanding but also fostered collaborative learning, a key component of effective medical education. To evaluate the impact of this hybrid teaching model, students underwent three forms of assessment. Knowledge acquisition was measured through a post-training multiple-choice questionnaire (MCQ) that included ten questions related to the procedural steps, clinical indications and technical interpretation of the skills taught. Students were given ten minutes to complete the test, ensuring a focused evaluation of theoretical understanding. Skill acquisition was assessed through an objective structured practical examination (OSPE), where students were required to demonstrate their competency in front of an examiner. A standardized checklist was used to ensure objective evaluation, providing a quantifiable measure of procedural accuracy. Lastly, the perception of both students and faculty regarding the hybrid learning model was collected using a Likert-scale questionnaire. This survey captured insights into engagement, clarity, effectiveness and overall satisfaction with the teaching approach. By integrating stepwise skill-building with structured feedback and peer teaching, this hybrid learning model provided an interactive and evidence-based approach to competency-based medical education. The structured methodology ensured that students not only learned how to perform a procedure but also understood its broader clinical applications. Through a combination of hands-on training, critical reasoning and real-time feedback, this approach aimed to bridge the gap between theoretical knowledge and practical execution, ultimately enhancing students' confidence and competency in performing clinical procedures. Future research should explore long-term skill retention and the potential application of this model to more advanced medical training scenarios (Table 2, Table 3, Table 4).

## Discussion:

This study revealed that the hybrid teaching model, which integrates the "So-to-Do" technique with the modified one-minute preceptor (OMP) model, significantly enhanced student performance. The structured nature of this approach ensured that students developed both theoretical knowledge and practical skills, making them more confident in their learning process. The hybrid method, which combines stepwise skill development, direct observation, supervised practice, structured feedback and peer teaching, allowed students to transition smoothly from theory to hands-on application. By incorporating real-time correction and reinforcement, the model helped students gain a deeper understanding of concepts while improving their procedural accuracy. This aligns well with the competency-based medical education (CBME) framework, which emphasizes both cognitive and psychomotor learning to create well-rounded medical professionals [36, 37]. The results of the study provided concrete of the model's effectiveness. The knowledge assessment scores revealed that students trained using the hybrid approach achieved an average faculty-assigned score of 5.26 ± 1.68 out of 10, with a coefficient of variation (CV) of 32% [38]. Similarly, in the objective structured practical examination (OSPE) for skill assessment, students demonstrated an identical mean score of 5.26 ± 1.68, again with a CV of 32%, suggesting that both knowledge acquisition and skill performance were enhanced at the same level [38]. This consistency highlights how structured learning phases, immediate feedback and supervised practice contribute to balanced educational outcomes. The ability to not just memorize procedural steps but also understand the reasoning behind them played a key role in ensuring better retention and accuracy during clinical practice. Students also benefitted from real-time feedback, which allowed them to correct mistakes instantly and refine their techniques, leading to increased procedural confidence [39, 40].

Our study findings align with existing research on hybrid learning models in medical education. Vallée *et al.* (2020) concluded that blended learning significantly outperformed traditional lecture-based teaching in improving knowledge retention and skill application among medical students [36]. Similarly, Edafe *et al.* found that progressive teaching programs grounded in the Fairness principles-Feedback, Activity, Individuality, and Relevance-can serve as an effective model for enhancing undergraduate clinical education [37]. Another study by Buckley *et al.* reported that making portfolios may help students reflect and improve learning, but more strong studies are needed to prove their real benefits [38]. The results of these studies reinforce the importance of structured, blended approaches that balance theoretical and hands-on learning, fostering deeper understanding and skill mastery [39, 40]. However, not all studies unanimously support hybrid teaching models. He *et al.* (2020) found no statistically significant difference in knowledge acquisition between hybrid and traditional teaching methods in medical education [39]. Additionally, Pai *et al.* (2022) observed that students who underwent online-only clinical examination skills training scored significantly lower in OSPE assessments compared to their peers who attended in-person training [40]. These findings highlight a crucial point-while hybrid learning enhances theoretical knowledge and engagement, practical skills training still benefits significantly from face-to-face interactions. This aligns with our study's results, emphasizing that hands-on practice remains a fundamental component of effective skill acquisition in medical education [41, 42].

Both students and faculty expressed high levels of satisfaction with the hybrid model. Students appreciated the engaging, interactive and structured nature of the training, which they felt provided a more practical and application-based understanding compared to traditional classroom methods [38]. Faculty members also acknowledged the model's ability to promote critical thinking, stepwise reinforcement and real-time feedback, making it a more effective way to train medical students in clinical skills [39]. This sentiment is supported by Kausar *et al.* (2023), who found that 66% of MBBS students preferred hybrid learning over purely in-person or online-only instruction [41].

Flipped classroom learning enhances cognitive performance and student engagement in anatomy education [42]. Research suggests that simulation-based hybrid models lead to higher procedural confidence and improved OSPE performance among medical students [43]. Blended learning integrating MOOCs and case-based learning enhances both performance and motivation in medical pathophysiology education [44]. Furthermore, a 2024 meta-analysis on flipped classrooms in medical education confirmed that hybrid models consistently outperformed traditional methods in knowledge retention, engagement and hands-on skill acquisition [45]. The external validation of our hybrid model by medical education experts reinforces its potential for broader implementation in competency-based curricula [46].

## Limitations and future directions:

While this study provides strong evidence supporting the effectiveness of the hybrid teaching model, it is important to acknowledge its limitations. One key limitation is the relatively small sample size, as the study was conducted within a single institution. This means that while the results are promising, they may not be entirely generalizable to larger or more diverse student populations. To gain a more comprehensive understanding of the model's impact, future studies should expand to multiple institutions and involve a larger cohort of students. Another important factor to consider is long-term skill retention. This study primarily assessed student performance in the short term, evaluating immediate knowledge and skill acquisition. However, it remains unclear how well students retain and apply these skills months or years down the line. Future research should include longitudinal follow-up studies to track how well students continue to perform in clinical settings over time. Additionally, assessing how this model impacts real-world clinical decision-making and patient care would provide valuable insights into its broader effectiveness. Further refinement of the hybrid model is also needed to optimize its implementation in medical curricula. While students and faculty responded positively to the structured learning phases and interactive components, challenges such as faculty training, time constraints and resource availability must be addressed. Research should explore ways to integrate technology-driven tools, such as AI-driven assessments, virtual simulations and gamification techniques, to further enhance student engagement and personalized learning experiences [47, 48, 49-50].

## Conclusion:

The utilization of the SO-TO-DO method along with a modified OMP model creates enhanced medical educational value through its combination of organized feedback with practical hands-on training. Students together with faculty members indicated that their involvement and comprehension of clinical procedures developed in a positive direction. The hybrid implementation shows important potential to deliver theory and practice integration in competency-based learning.

## Figures and Tables

**Table 1:** Methodology of hybrid technology

**Observation Phase**	**Trainees begin by observing the procedure performed by the trainer. This phase allows trainees to familiarize themselves with the procedural workflow, contextual details and the subtleties involved in execution.**
Explanation Phase	Following observation, the trainer elucidates the procedure comprehensively. This phase includes an in-depth discussion of each procedural step, underlying principles, best practices, potential pitfalls and the rationale for specific actions.
Performance Phase	Trainees proceed to perform the procedure under the direct supervision of the trainer. This hands-on practice is critical for skill acquisition and the development of procedural confidence.
Critical Reasoning and Information Retrieval	During the performance phase, the trainer engages the trainee by querying critical information and the reasoning behind each procedural step. This dialogue reinforces theoretical knowledge and its application in a practical context.
Feedback Phase	The trainer provides constructive feedback, highlighting successful execution and addressing any errors. Immediate correction of mistakes ensures that trainees internalize the correct techniques and avoid repetition of errors.
Peer Teaching Phase	Trainees then instruct other trainees on the procedure, thereby reinforcing their own knowledge and skills through the act of teaching. This peer-teaching component also includes the identification and correction of any observed errors.
Hybrid Model Integration	Our developed hybrid model integrated the “See One, Teach One, Do One” (SO+TO+DO) method with the modified One-Minute Preceptor (OMP) method. These hybrid approaches synergistically combined the observational and practical components of SO+TO+DO with the structured feedback and critical reasoning elements of the OMP method.

**Table 2:** Knowledge assessments with various teaching learning tools

**Knowledge Assessment**		
T/L Tool	Weighted grade awarded by faculty (out of 10) (mean ± SD)	CV (%)
HYBRID MODEL	5.26 ± 1.68	32%

**Table 3:** Skill assessment through OSPE

**Skill assessment**		
T/L Tool	Weighted grade awarded by faculty (out of 10) (mean ± SD)	CV (%)
HYBRID MODEL	5.26 ± 1.68	32%

**Table 4:** Comparison of present study findings with previous research

**Study**	**Type**	**Knowledge Score (Mean ± SD)**	**OSPE Score (Mean ± SD)**	**Key Finding**	**Similarity/Difference**
Current Study (2025)	126 MBBS students	5.26 ± 1.68	5.26 ± 1.68	Hybrid model improved both knowledge and skill acquisition	Baseline study for comparison
Vallée *et al.* (2020) [36]	Meta-analysis	Higher than traditional learning	Higher than traditional learning	Hybrid learning improved knowledge retention and skills	Similar - Supports effectiveness of hybrid learning
Singh *et al.* (2024) [37]	RCT in anatomy	Higher than traditional methods	Higher than traditional methods	Hybrid model increased knowledge and OSPE performance	Similar - Reinforces structured hybrid training
Parthasarthy *et al.* [2024] [38]	120 MBBS students	Higher than control	Higher than control	OSPE-integrated hybrid training enhanced practical skills	Similar - Confirms benefit of OSPE inclusion
He *et al.* (2020) [39]	Systematic review	No significant difference	No significant difference	No clear advantage of hybrid over traditional methods	Dissimilar - Contradicts current findings
Pai *et al.* (2022) [40]	Clinical skills training	Lower than in-person training	Lower than in-person training	Fully online training led to weaker skill acquisition	Dissimilar - Supports need for in-person components
